# Development of a prediction model based on LASSO regression to evaluate the risk of non-sentinel lymph node metastasis in Chinese breast cancer patients with 1–2 positive sentinel lymph nodes

**DOI:** 10.1038/s41598-021-99522-3

**Published:** 2021-10-07

**Authors:** Lei Meng, Ting Zheng, Yuanyuan Wang, Zhao Li, Qi Xiao, Junfeng He, Jinxiang Tan

**Affiliations:** 1 Department of Endocrine and Breast Surgery, The First Affiliated Hospital of Chongqing Medical University, Chongqing, China; 2 Chongqing Traditional Chinese Medicine Hospital, Chongqing, China

**Keywords:** Cancer, Breast cancer, Oncology, Cancer, Surgical oncology

## Abstract

This study aimed to develop an intraoperative prediction model to evaluate the risk of non-sentinel lymph node (NSLN) metastasis in Chinese breast cancer patients with 1–2 positive sentinel lymph nodes (SLNs). The clinicopathologic data of 714 patients with 1–2 positive SLNs were investigated. Univariate and multivariate analyses were performed to identify the risk factors of NSLN metastasis. A new mathematical prediction model was developed based on LASSO and validated in an independent cohort of 131 patients. The area under the receiver operating characteristic curve (AUC) was used to quantify performance of the model. Patients with NSLN metastasis accounted for 37.3% (266/714) and 34.3% (45/131) of the training and validation cohorts, respectively. A LASSO regression-based prediction model was developed and included the 13 most powerful factors (age group, clinical tumour stage, histologic type, number of positive SLNs, number of negative SLNs, number of SLNs dissected, SLN metastasis ratio, ER status, PR status, HER2 status, Ki67 staining percentage, molecular subtype and P53 status). The AUCs of training and validation cohorts were 0.764 (95% CI 0.729–0.798) and 0.777 (95% CI 0.692–0.862), respectively. We presented a new prediction model with excellent clinical applicability and diagnostic performance for use by clinicians as an intraoperative clinical tool to predict risk of NSLN metastasis in Chinese breast cancer patients with 1–2 positive SLNs and make the final decisions regarding axillary lymph node dissection.

## Introduction

As the early diagnosis rate and systemic treatment of breast cancer are improving, breast cancer surgery is becoming less traumatic and more individualized. The axillary lymph node (ALN) status is one of the most critical prognostic factors in patients with breast cancer. In the last century, sentinel lymph node biopsy (SLNB) has been indicated to be a reliable method in the axilla stage^1^. Currently, axillary lymph node dissection (ALND) can be safely avoided in breast cancer patients with negative sentinel lymph nodes (SLNs)^2^. However, ALND remains the standard management strategy when breast cancer patients are determined to have positive SLNs^3^*.* In recent years, data from the American College of Surgeons Oncology Group Z0011 (ACOSOG Z0011) and the International Breast Cancer Study Group 23-01 (IBCSG 23-01) trials showed that further ALND did not result in additional benefit in terms of locoregional recurrence (LRR), disease-free survival (DFS) or overall survival (OS) in patients with limited SLN involvement^4,5^. However, most patients in these studies underwent breast-conserving surgery with whole-breast irradiation, and the ALND group included fewer patients with good prognostic features of a smaller tumour diameter, hormone receptor positivity, a lower number of positive SLNs, and the absence of lymphovascular invasion (LVI). In addition, information regarding the specific status of human epidermal growth factor receptor 2 (HER2) and KI67 and strict quality control of radiotherapy were lacking in these studies. In most developing countries, such as China, ALND is still recommended by most clinicians for patients with positive SLNs for the following reasons: (1) the differences between Eastern and Western populations; (2) a lack of evidence-based medical research in Eastern populations; (3) the uneven distribution of medical resources; and (4) the low rate of breast-conserving surgery (BCR) in China^6^. However, ALND is associated with increased morbidity, such as lymphoedema, paraesthesia and decreased mobility^7^. Moreover, clinical evidence has shown that only 40% of patients with positive SLNs have further axillary involvement, highlighting the importance of a prediction model to evaluate the risk of non-sentinel lymph node (NSLN) metastasis in breast cancer patients with 1–2 positive SLNs^8^. The Memorial Sloan Kettering Cancer Center (MSKCC) nomogram proposed by Van Zee et al. is the first model to predict NSLN metastasis in patients with positive SLNs. This nomogram contains the following nine independent variables: frozen section performed, pathological size, tumor type and grade, number of positive SLNs, SLN method of detection, number of negative SLNs, LVI, multifocality, and estrogen receptor(ER) status. This nomogram performed well in the training cohort (702 cases) and the validation cohort (373 cases) with AUCs of 0.76 and 0.77, respectively^9^. Other prediction models, such as the Louisville models, MD Anderson Cancer Center score and Tenon score, were developed based on Western populations^10–12^. A study conducted in Japan validated the performance of the MSKCC nomogram and Stanford nomogram in Asian populations. The results showed that the AUCs in macrometastasis and micrometastasis/ITC groups were 0.680 and 0.469 with the MSKCC nomogram and 0.676 and 0.574 with the Stanford nomogram, respectively, suggesting that these nomograms could not reliably predict the metastasis of non-sentinel lymph node in an Eastern population^13^. A prediction model designed specifically for the Eastern population is lacking. Therefore, we sought to develop a new intraoperative mathematical prediction model based on a Chinese population to evaluate the risk of NSLN metastasis in Chinese breast cancer patients with 1–2 positive SLNs. Least absolute shrinkage and selection operator (LASSO) regression was used to construct the model.

## Methods

### Patients

Patients were included in our study according to the following eligibility criteria: (1) diagnosis of primary breast cancer; (2) no signs of ALN involvement discernible by physical examination or imaging; (3) the presence of only one or two positive SLNs; (4) treatment with further ALND; and (5) the availability of complete clinicopathological data. We excluded patients who had undergone neoadjuvant therapy, patients with inflammatory breast cancer or bilateral breast cancer, and patients with a history of breast cancer. The clinicopathological data of breast cancer patients who underwent SLNB and ALND at The First Affiliated Hospital of Chongqing Medical University between January 2013 and December 2018 were retrospectively collected and analysed. A mathematical prediction model was developed. The data of patients treated between January 2019 and December 2019 were used for validation.

### Surgical procedures

SLNB was performed by injecting a radiocolloid (^99m^Tc) combined with methylene blue. First, the ^99m^Tc-labelled sulfur colloid (Xinke, Beijing, China) was injected into the subareolar area 3–18 h before surgery. Then, the surgeon injected methylene blue (Jumpcan, Nanjing, China) into the subareolar area and massaged the breast for 5–10 min before the operation. Lymph nodes, any blue-stained nodes and nodes with a high radioactive count (at least 10%) as measured by a gamma detector (neo2000^@^, Johnson & Johnson, US) were classified as SLNs and removed for the preparation of intraoperative frozen sections. All clinicopathological data were collected from preoperative biopsy and intraoperative frozen sections. All SLNs and ALNs dissected during surgery were routinely submitted for postoperative pathological sections and immunohistochemical (IHC) staining.

### Diagnostic criteria

The tumours were classified by size as T1, T2 and T3 according to the 8th edition of the American Joint Committee on Cancer (AJCC) staging guidelines^14^. IHC staining was performed to determine the estrogen receptor (ER), progesterone receptor (PR) and Ki67 status. The HER2 status was determined by IHC staining combined with fluorescence in situ hybridization (FISH). The samples that were IHC (−) and IHC (1+) were considered HER2-negative, and the samples that were IHC (3+) and FISH (+) were considered HER2-positive. All cases in the study were classified as the luminal A, luminal B, HER2 overexpression or Triple negative subtype by the 2013 St Gallen International Expert Consensus^15^.

### Statistical analysis

The χ^2^ test and logistic regression were utilized for the univariate and multivariate analyses, respectively. The analyses were performed using SPSS 23.0 software. The LASSO regression was performed using the glmnet package in R version 3.6.2 to establish a mathematical prediction model calculating the risk scores (RS) of the patients. The RS of each patient was calculated by the mathematical formula. We used SPSS 23.0 software to generate the receiver operating characteristic (ROC) curve. The performance of the prediction model was assessed by the area under the ROC curve (AUC) in the training cohort and the validation cohort. *P* < 0.05 was considered significant.

### Ethical approval and consent to participate

This study was approved by the ethics committee of the First Affiliated Hospital of Chongqing Medical University (2020-309), and each participating patient provided written informed consent. All methods were confirmed to be performed in accordance with the relevant guidelines and regulations.

## Results

### General demographic and characteristics

Ultimately, in total, 845 patients with 1–2 positive SLNs were enrolled in our study; 714 patients were included in the training cohort and 131 patients were included in the validation cohort. All patients were females who ranged in age from 22 to 88 years. The median age was 49 years in both groups. In the training cohort, 266 patients (37.3%) were demonstrated to have NSLN metastasis by an analysis of postoperative pathological sections, indicating that the other 448 patients (62.7%) underwent unnecessary ALND. Then, the patients in the training cohort were divided into the low-RS group and the high-RS group, in which 26 patients (10.8%) and 240 patients (50.6%), respectively, were observed to have further NSLN metastasis. Additional clinicopathological parameters in the training cohort are shown in Table 1. The clinicopathological characteristics of patients in the validation cohort are shown in Table 2.

**Table 1:** Clinicopathological characteristics of the 714 patients in the training cohort.

Variable	NSLN-negative (%) (N = 448)	NSLN-positive (%) (N = 266)	*χ*^2^	*P*
**Age group (years)**			0.958	0.328
≤ 50	271 (64.2)	151 (35.8)		
> 50	177 (60.6)	115 (39.4)		
**Side**			0.273	0.602
Left	228 (63.7)	130 (36.3)		
Right	220 (61.8)	136 (38.2)		
**Location**			2.698	0.610
Centre area	16 (53.3)	14 (46.7)		
Upper inner quadrant	84 (67.7)	40 (32.3)		
Lower inner quadrant	49 (60.5)	32 (39.5)		
Upper outer quadrant	180 (62.1)	110 (37.9)		
Under outer quadrant	119 (63.0)	70 (37.0)		
**Clinical tumour stage**			3.617	0.164
T1	220 (66.3)	112 (33.7)		
T2	224 (59.9)	150 (40.1)		
T3	4 (50.0)	4 (50.0)		
**Histologic type**			2.494	0.287
Invasive ductal carcinoma	426 (62.2)	259 (37.8)		
Invasive lobular carcinoma	9 (81.8)	2 (18.2)		
Other	13 (72.2)	266 (37.3)		
**Histologic grade**			9.204	0.010
I	17 (70.8)	7 (29.2)		
II	377 (64.8)	205 (35.2)		
III	54 (50.0)	54 (50.0)		
**Number of positive SLNs**			26.285	< 0.001
1	347 (68.7)	158 (31.3)		
2	101 (48.3)	108 (51.7)		
**Number of negative SLNs**			97.841	< 0.001
0	58 (36.5)	101 (63.5)		
1	77 (50.0)	77 (50.0)		
≥ 2	313 (78.1)	88 (21.9)		
**Number of SLNs dissected**			68.321	< 0.001
1–2	110 (42.8)	147 (57.2)		
≥ 3	338 (74.0)	119 (26.0)		
**SLN metastasis ratio**			105.242	< 0.001
< 0.5	299 (80.6)	72 (19.4)		
≥ 0.5	149 (43.4)	194 (56.6)		
**LVI**			16.638	< 0.001
No	442 (64.2)	247 (35.8)		
Yes	6 (24.0)	19 (76.0)		
**ER**			6.536	0.011
Negative	92 (54.4)	77 (45.6)		
Positive	356 (65.3)	189 (34.7)		
**PR**			3.323	0.068
Negative	137 (58.1)	99 (41.9)		
Positive	311 (65.1)	167 (34.9)		
**HER2**			7.787	0.005
Negative	326 (66.1)	167 (33.9)		
Positive	122 (55.2)	99 (44.8)		
**Ki67 (%)**			2.027	0.155
< 14	165 (66.3)	84 (33.7)		
≥ 14	283 (60.9)	182 (39.1)		
**Molecular subtype**			16.043	0.001
Luminal A	102 (68.0)	48 (32.0)		
Luminal B	263 (64.9)	142 (35.1)		
HER2 overexpression	37 (43.5)	48 (56.5)		
Triple negative	46 (62.2)	28 (37.8)		
**P53**			0.031	0.860
Negative	142 (62.3)	86 (37.7)		
Positive	306 (63.0)	180 (37.0)		
RS*			107.966	< 0.001
Low	214 (89.2)	26 (10.8)		
High	234 (49.4)	240 (50.6)		

*NSLN* non-sentinel lymph node, *SLNs* sentinel lymph nodes, *LVI* lymphovascular invasion, *ER* estrogen receptor, *PR* progesterone receptor, *HER2* human epidermal growth factor receptor 2, *RS* risk score.

*The patients were divided into the Low-RS group and High-RS group when the cut-off value of RS was set as 1.059328005.

**Table 2:** Clinicopathological characteristics of the 131 patients in the validation cohort.

Variable	Validation cohort (%) (N = 131)
**NSLN**	
Positive	45 (34.3)
Negative	86 (65.7)
**Age group (years)**	
≤ 50	71 (54.2)
> 50	60 (45.8)
**Side**	
Left	58 (44.3)
Right	73 (55.7)
**Location**	
Centre area	11 (8.4)
Upper inner quadrant	24 (18.3)
Lower inner quadrant	10 (7.6)
Upper outer quadrant	67 (51.2)
Under outer quadrant	19 (14.5)
**Clinical tumour stage**	
T1	45 (34.3)
T2	83 (63.4)
T3	3 (2.3)
**Histologic type**	
Invasive ductal carcinoma	125 (95.4)
Invasive lobular carcinoma	4 (3.1)
Other	2 (1.5)
**Histologic grade**	
I	2 (1.5)
II	103 (78.6)
III	26 (19.9)
**Number of positive SLNs**	
1	81 (61.8)
2	50 (38.2)
**Number of negative SLNs**	
0	26 (19.8)
1	19 (14.5)
≥ 2	86 (65.7)
**Number of SLNs dissected**	
1–2	37 (28.2)
≥ 3	94 (71.8)
**SLN metastasis ratio**	
< 0.5	76 (58.0)
≥ 0.5	55 (42.0)
**LVI**	
No	117 (89.3)
Yes	14 (10.7)
**ER**	
Negative	26 (19.8)
Positive	105 (80.2)
**PR**	
Negative	37 (28.2)
Positive	94 (71.8)
**HER2**	
Negative	99 (75.6)
Positive	32 (24.4)
**Ki67 (%)**	
< 14	34 (26.0)
≥ 14	97 (74.0)
**Molecular subtype**	
Luminal A	31 (23.7)
Luminal B	75 (57.2)
HER2-enriched	8 (6.1)
Triple negative	17 (13.0)
**P53**	
Negative	57 (43.5)
Positive	74 (56.5)

### Univariate and multivariate analyses in the training cohort

The univariate analysis showed that the histologic grade (*P* = 0.010), number of positive SLNs (*P* < 0.001), number of negative SLNs (*P* < 0.001), number of SLNs dissected (*P* < 0.001), SLN metastasis ratio (*P* < 0.001), LVI status (*P* < 0.001), ER status (*P* = 0.011), HER2 status (*P* = 0.005), molecular subtype (*P* = 0.001), and RS (*P* < 0.001) were associated with NSLN involvement in breast cancer patients with 1–2 positive SLNs (Table 1). In further logistic regression multivariate analysis, the histologic grade (*P* = 0.026), LVI status (*P* = 0.005), number of positive SLNs (*P* = 0.001), number of negative SLNs (*P* = 0.005), SLN metastasis ratio (*P* = 0.005), and molecular subtype (*P* = 0.007) were identified as independent predictive factors for NSLN metastasis (Table 3). Compared with the triple negative breast cancer patients, patients with the HER2 overexpression subtype were more likely to have positive NSLNs, whereas the patients with the luminal A and luminal B subtypes did not significantly differ in NSLN metastasis.

**Table 3:** Multivariate analysis of variables associated with NSLN metastasis.

Variables	*OR*	95% *CI*	*P*
LVI	4.378	1.569–12.214	0.005
Histologic grade	1.630	1.061–2.505	0.026
Number of positive SLNs	1.898	1.299–2.773	0.001
Number of negative SLNs	0.594	0.414–0.853	0.005
SLN metastasis ratio	2.414	1.307–4.459	0.005
Molecular subtype			0.007
Triple negative	1	Reference	
Luminal A	0.690	0.352–1.351	0.279
Luminal B	0.873	0.487–1.565	0.649
HER2 overexpression	1.990	0.972–4.071	0.060

### Development of a mathematical prediction model based on LASSO regression

**Figure 1:**
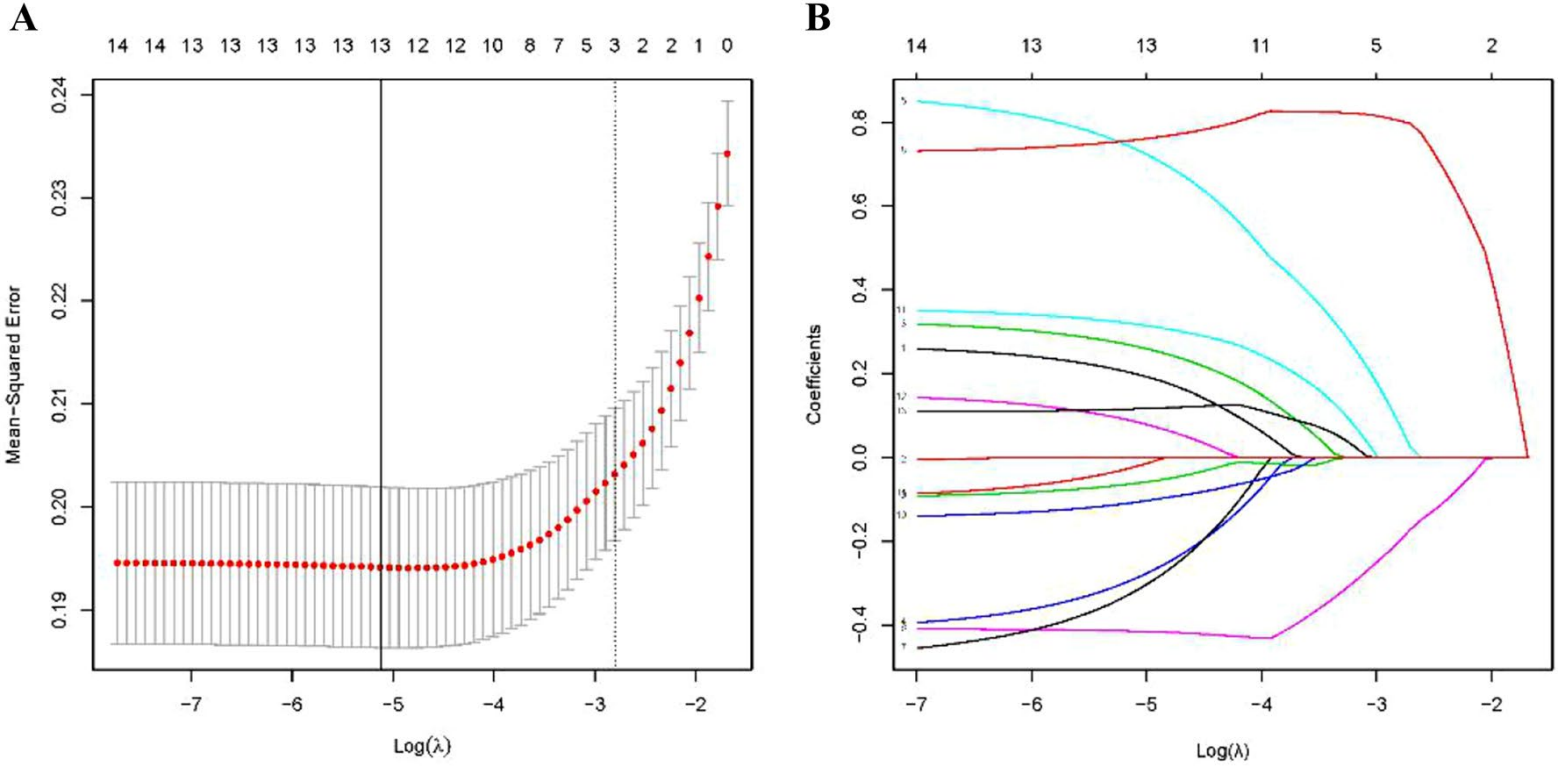
Identification of the influencing factors by LASSO regression. LASSO regression identified the following 13 most powerful predictors: (**A**) age group, clinical tumour stage, histologic type, number of positive SLNs, number of negative SLNs, number of SLNs dissected, SLN metastasis ratio, ER status, PR status, HER2 status, Ki67 staining percentage, molecular subtype and P53 status. Plot (**B**) shows the coefficients of each predictor when the 13 predictors were included in the LASSO regression model.

In our study, LASSO regression was used to develop a mathematical prediction model. Finally, the LASSO regression analysis identified the following 13 most powerful factors: the age group, clinical tumour stage, histologic type, number of positive SLNs, number of negative SLNs, number of SLNs dissected, SLN metastasis ratio, ER status, PR status, HER2 status, Ki67 staining percentage, molecular subtype and P53 status (Fig. 1). Among these factors, the SLN metastasis ratio was the most influential factor in the RS, with the maximum absolute value of the coefficient. The regression coefficients of each factor are shown in Table 4. The RS of each patient in the study was calculated using the following model equation:

$$ {\text{RS}} =\upbeta _{1} {\text{X}}_{1} +\upbeta _{2} {\text{X}}_{2} + \cdots +\upbeta _{{\text{n}}} {\text{X}}_{{\text{n}}} \left( {{\text{X}}:{\text{a\;factor}};\upbeta :{\text{the\;regression\;coefficient\;of\;that\;factor}}} \right). $$

**Table 4:** Regression coefficients of the 13 most powerful factors identified by the LASSO regression analysis.

Factors	LASSO coefficient
Age group	0.1799442245
Clinical tumour stage	0.2484027572
Histologic type	− 0.2555910122
Number of positive SLNs	0.7014400762
Number of negative SLNs	− 0.4169544368
Number of SLNs dissected	− 0.2759444450
SLN metastasis ratio	0.7672377992
ER	− 0.0520947933
PR	− 0.0962431976
HER2	0.3078328089
Ki67	0.0675226829
Molecular subtype	0.1188944693
P53	− 0.0006942997

### Performance of the prediction model

**Figure 2:**
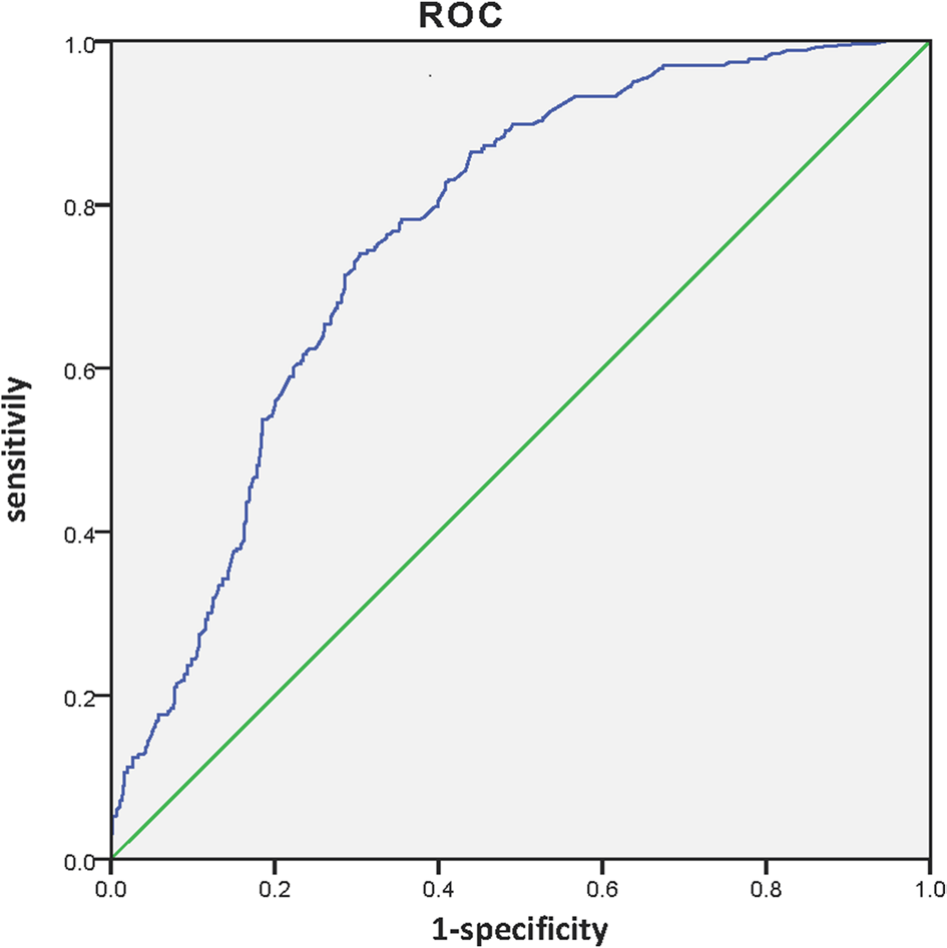
ROC curve in the training cohort. The AUC was 0.764 (95% CI 0.729–0.798).

**Figure 3:**
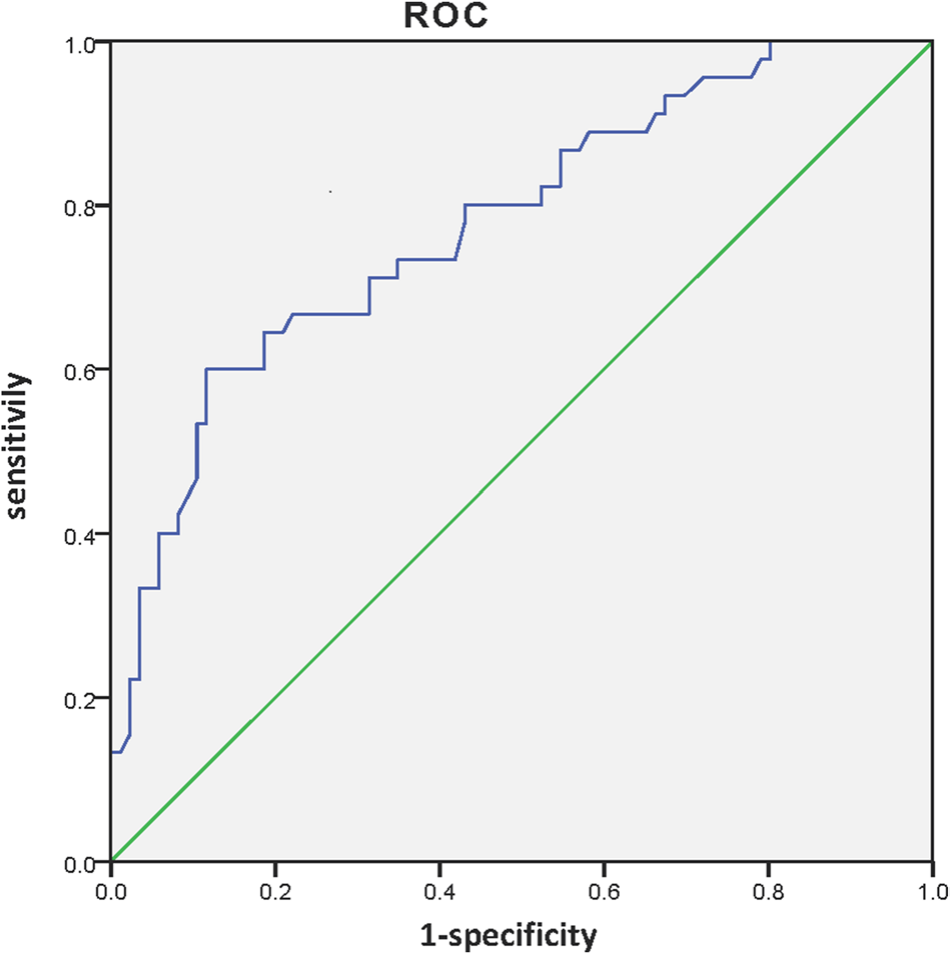
ROC curve in the validation cohort. The AUC was 0.777 (95% CI 0.692–0.862).

Subsequently, we generated the ROC curve of the prediction model. The AUC was 0.764 (95% CI 0.729–0.798), highlighting the excellent diagnostic performance of this model (Fig. 2). The RS value of 1.87239924305 was identified as the cut-off value with the highest Youden index. The sensitivity, specificity and overall accuracy of the model were 74.1%, 69.6% and 71.3%, respectively. More than 30% of the patients in the study would avoid unnecessary ALND with the prediction model. In the validation cohort of 131 patients, the AUC was 0.777 (95% CI 0.692–0.862) (Fig. 3), showing a satisfying predictive value.

## Discussion

In recent years, the results of the ACOSOG Z0011 and IBCSG 23-01 trials showed that neither the DFS nor the OS significantly differed between the SLNB-only and ALND groups among breast cancer patients with limited SLN involvement. Based on the results of these trials, the latest NCCN guidelines also recommended not performing ALND in patients with 1–2 involved SLNs who are planning to undergo breast-conserving surgery and subsequent radiotherapy^16^. However, in most developing countries, such as China, the BCR is only approximately 20%, while that in Western countries is 50–80%^6,17,18^. Even in some leading centres in China, the BCR is only 30%^19^. Because of the low BCR in developing countries and the absence of evidence of ALND omission in Eastern populations, most clinicians in developing countries such as China still hold a conservative view and recommend ALND for patients with positive SLNs^17^. In addition, the ALN status remains one of the most important prognostic factors. In our present study, only 266 (37.3%) of the patients with 1–2 SLN metastases in the training cohort were demonstrated to have NSLN metastasis after ALND, which is consistent with the results of previous studies^8,20^. Thus, more than 60% of the patients received unnecessary ALND. Therefore, it is of great importance to accurately predict NSLN metastasis either intraoperatively or preoperatively. Our study retrospectively analysed the clinicopathological data of the 714 breast cancer patients with 1–2 positive SLNs in the training cohort to determine the factors associated with axillary involvement. We further developed a new mathematical prediction model based on this Chinese population to evaluate the risk of NSLN metastasis.

Previous studies have shown that LVI is a feature related to a poor prognosis and that promotes local recurrence and distant metastasis of tumours^21^. Several recent studies have recognized LVI as an independent predictor of NSLN metastasis in patients with 1–2 positive SLNs^22,23^. We drew the same conclusion. In our study, 76.0% and 35.8% of the patients with LVI and without LVI, respectively, were found to have NSLN involvement, and this difference was significant. Whether histologic grade is associated with NSLN metastasis remains controversial, and the conclusions reported by Maimaitiaili A and Wang XY are inconsistent^24,25^. Our univariate analysis showed that the patients with higher histologic grades were more likely to have at least one positive ALN, and the histologic grade remained an independent predictor of NSLN metastasis in the subsequent multivariate analysis (OR 1.630; 95% CI 1.061–2.505; *P* = 0.026).

Moreover, we divided all patients in the training cohort into the luminal A, luminal B, HER2 overexpression and triple negative subtypes according to the St Gallen International Expert Consensus (2013 edition)^15^. In these respective molecular subtype groups, 32.0%, 35.1%, 56.5% and 37.8% of the patients exhibited NSLN involvement. Compared to the triple negative type, the HER2 overexpression type, but not the luminal A and luminal B subtypes, was associated with a statistically higher risk of positive NSLNs. Whether NSLN metastasis is associated with the molecular subtype remains controversial. The results of a recent single-centre study involving 291 patients demonstrated that patients with luminal B and HER2 overexpression breast cancer had a significantly higher possibility of having at least one positive NSLN than patients with luminal A breast cancer^26^. However, in another retrospective study, investigators failed to identify the molecular subtype as an independent predictor of NSLN metastasis. The patients with positive SLNs had the same risk of axillary involvement regardless of their molecular subtypes^22^.

The number of positive SLNs, number of negative SLNs, number of SLNs dissected and SLN metastasis ratio were important predictors of NSLN metastasis in breast cancer patients with 1–2 positive SLNs. These factors heavily rely on assessments of intraoperative frozen sections. Thus, these values are unclear prior to surgery. Two publications considered the numbers of positive and negative SLNs independent risk factors and included these factors in their prediction models^27,28^. The SLN metastasis ratio was incorporated into the model predictions in another clinical study^29^. However, the value of the number of positive SLNs, number of negative SLNs, number of SLNs dissected and SLN metastasis ratio in predicting NSLN metastasis has not been fully clarified because of the collinearity among these factors. In our study, a LASSO regression was used to construct the mathematical model, thereby effectively solving the problem of collinearity among these factors.

The MSKCC nomogram, which was the first model to predict NSLN metastasis in patients with positive SLNs, performed well in the original population, with an AUC of 0.76^9^. The results of a previous study showed that the AUC of the MSKCC nomogram was less than 0.7, proving that the performance of the MSKCC nomogram was inferior to that of other models in other populations^30–32^. In addition, the MSKCC nomogram and other previous models were developed based on Western populations in developed countries, and thus hardly apply to the Eastern population. In the present retrospective analysis, we developed a new, LASSO algorithm-based intraoperative mathematical prediction model based on the clinical data of 714 patients in China for evaluating the risk of NSLN metastasis in Chinese breast cancer patients with 1–2 positive SLNs. The LASSO algorithm forces the sum of the absolute value of the regression coefficients to be less than a fixed value by reducing certain coefficients to zero, which helps to effectively construct a simpler model that includes only the most meaningful predictive factors. Ultimately, the LASSO regression identified the following 13 most powerful predictors: age group, clinical tumour stage, histologic type, number of positive SLNs, number of negative SLNs, number of SLNs dissected, SLN metastasis ratio, ER status, PR status, HER2 status, Ki67 staining percentage, molecular subtype and P53 status. The coefficients of each predictor are shown in Table 4. The higher the absolute value of a regression coefficient, the greater is its influence on the model. In our prediction model, the most powerful predictor was the SLN metastasis ratio, with a coefficient of 0.7672377992. This factor was also included in the Cambridge model and another recent model^33,34^.

Previous evidence showed that the absolute agreement rates of histologic grade and LVI between specimens obtained by core needle biopsy (CNB) and those obtained by surgical excision were only 75% and 69%, respectively^35,36^. The small number of specimens from CNB and intratumoural heterogeneity are possible reasons for the low concordance rate of the histologic grade and LVI status between the preoperative CNB and postoperative pathology results. Considering that the aim of our study was to develop an intraoperative prediction model, the histologic grade and LVI status, which are not entirely available via preoperative or intraoperative evaluation, were excluded from our prediction model, although these factors were identified as independent risk factors in the retrospective multivariate analysis.

Furthermore, we calculated the RS of each patient in the training cohort according to the model equation. The ROC curve of the prediction model was then generated and shown to have an AUC of 0.764 (95% CI 0.729–0.798), which is comparable to that of the MSKCC nomogram^9^. Thus, the predictive power of the model is acceptable. Finally, the ROC curve analysis confirmed the cut-off value of the RS to be 1.87239924305. The sensitivity, specificity and total accuracy were 74.1%, 69.6% and 71.3%, respectively. More than 30% of the patients in the study would avoid unnecessary ALND with the prediction model. Therefore, we believe that ALND may be safely ignored when the RS of a patient, as calculated by the model equation, is less than the cut-off value of 1.87239924305. We divided the patients in the training cohort into a low-RS group and a high-RS group according to the cut-off value. Significantly more patients had positive NSLNs in the high-RS group than in the low-RS group, further confirming the predictive power of our model.

To evaluate the clinical applicability of the prediction model, a subsequent independent cohort of 131 patients was used for validation. The model still showed impressive performance, with an AUC of 0.777 (95% CI 0.692–0.862). Tus, the present prediction model can be considered an intraoperative clinical tool for clinicians to predict the risk of NSLN metastasis in Chinese breast cancer patients with 1–2 positive SLNs and make decisions regarding ALND.

To the best of our knowledge, this study is the first to use the LASSO algorithm to develop a prediction model based on an Eastern population. As more than 13 factors were included in our model, this model offers a more personalized assessment for breast cancer patients. However, there are a few limitations in our study. First, this was a retrospective study, and a prospective clinical trial is greatly needed. Second, this study was a single-centre study. Our prediction model should be validated with population data from other centres.

## Conclusion

In conclusion, we developed a new intraoperative mathematical prediction model with 13 predictors based on the LASSO algorithm to evaluate the risk of NSLN metastasis in Chinese breast cancer patients with 1–2 positive SLNs. The model performed well in both the training cohort and validation cohort and has good clinical applicability.

## Data Availability

The datasets supporting the findings of this study will be available from the corresponding author upon reasonable request.

## Abbreviations

ALN: Axillary lymph node
SLNB: Sentinel lymph node biopsy
ALND: Axillary lymph node dissection
SLNs: Sentinel lymph nodes
ACOSOG Z0011: American College of Surgeons Oncology Group Z0011
IBCSG 23-01: International Breast Cancer Study Group 23-01
LRR: Locoregional recurrence
DFS: Disease-free survival
OS: Overall survival
LVI: Lymphovascular invasion
HER2: Human epidermal growth factor receptor-2
BCR: Rate of breast-conserving surgery
NSLN: Non-sentinel lymph node
MSKCC: Memorial Sloan Kettering Cancer Center nomogram
LASSO: Least absolute shrinkage and selection operator
IHC: Immunohistochemical
AJCC: American Joint Committee on Cancer
ER: Estrogen receptor
PR: Progesterone receptor
FISH: Fluorescence in situ hybridization
RS: Risk scores
ROC: Receiver operating characteristic
AUC: Area under the ROC curve
CNB: Core needle biopsy
